# Stigma and contact with mental illness in a university population through volunteering: a case-control study

**DOI:** 10.1192/j.eurpsy.2023.557

**Published:** 2023-07-19

**Authors:** A. Madoz-Gúrpide, E. Ochoa Mangado, P. Cuadrado del Rey

**Affiliations:** ^1^Psychiatry Service, Ramón y Cajal University Hospital; ^2^ Alcalá University; ^3^Ramón y Cajal Institute for Health Research (IRYCIS), MADRID, Spain

## Abstract

**Introduction:**

Stigma in mental illness has a negative impact on the daily functioning of the patient, their personal development and their clinical prognosis. Direct contact with people who suffer from this pathology could modify the stigma towards these populations.

**Objectives:**

The objective of the study is to assess whether the stigma of mental illness in university students is modified by contact with people suffering from mental illness, established through volunteering activities with that population.

**Methods:**

Observational case-control study. The sample is made up of young subjects (18 to 35 years old) who have studied or are studying a university degree during the 2021-2022 academic year. The cases (n=91) are subjects who have ever volunteered with people diagnosed with mental illness. Those who have not had this experience constitute the control group (n=237).

The variables were collected by completing an anonymous online questionnaire. To analyze stigma, the Attribution Questionnaire-27 questionnaire was used, which offers a total score as well as 9 domains related to stigma. Statistical analysis (including multiple linear regression) was performed with the statistical package IBM SPSS Statistics, version 20.

**Results:**

Once adjusted for age and gender, the case group scores lower, with statistically significant differences, in the subscales Anger (p-value: 0.001), Dangerousness (p-value: 0.000), Fear (p-value: 0.000 ), Coercion (p-value: 0.028), Segregation (p-value: 0.000), Avoidance (p-value: 0.000), as well as in the Total Score (p-value: 0.000). Likewise, it is also observed that the group of cases score higher on the Help subscale (p-value: 0.002).

**Table:** 

**Coefficients**								
Model	Unstandardized Coefficients	Standardized Coefficients	t	Sig.	95% Confidence Interval for B			
	B	Std. Error	Beta			Lower limit		Upper Limit
	(Constant)	72,745	10,931		6,655	,000	51,234	94,256
	Volunteering	13,100	3,196	,236	4,098	,000	6,810	19,391
	Age	,669	,342	,113	1,956	,051	-,004	1,342
	Gender	-,196	2,941	-,004	-,067	,947	-5,983	5,591

a. Dependent Variable: Total Score

**Conclusions:**

Previous contact with patients with mental illness through voluntary activities seems to favor less stigma towards mental pathology.

**Disclosure of Interest:**

None Declared

